# Alexithymia In Multiple Sclerosis: Relationship With Depression

**DOI:** 10.1192/j.eurpsy.2022.1160

**Published:** 2022-09-01

**Authors:** M. Ben Abdallah, I. Baati, A. Zouari, F. Guermazi, S. Hentati, N. Farhat, J. Masmoudi

**Affiliations:** 1 CHU Hedi CHaker hospital Sfax Tunisia, Department Of Psychiatry (a), Sfax, Tunisia; 2 Habib Bourguiba University Hospital, Department Of Neurology, Sfax, Tunisia

**Keywords:** multiple sclerosis, Depression, alexithymia

## Abstract

**Introduction:**

Alexithymia, the lack of words to express emotions, is a common problem in multiple sclerosis (MS) patients.

**Objectives:**

To investigate the prevalence of alexithymia in patients with MS and to evaluate the factors related to it, including depression.

**Methods:**

We conducted a cross-sectional, descriptive and analytical study, which took place in the neurology department in Sfax (Tunisia). It involved MS outpatients in remission phase. Data collection was done using a form exploring sociodemographic, clinical and radiological characteristics. We used the Expanded Disability Status Scale (EDSS) to evaluate neurological impairments, the Toronto Alexithymia Scale (TAS-20) to assess alexithymia, and the Hospital Anxiety and Depression Scale (HADS) to assess depressive symptoms.

**Results:**

Our study included 93 patients. They were married in 57% of cases. The total number of relapses ranged from 1 to 30, with a median of 5. The EDSS score ranged from 0 to 8. A temporal lesion on brain imaging was found in 29% of cases. MS patients had alexithymia in 58.1% of cases and depression in 26.9% of cases. Alexithymia was more frequent in unmarried patients (p = 0.028). Among clinical and radiological factors, the number of relapses was higher (p = 0.035), and temporal lesion was more frequent in alexithymic patients (p = 0.045). In this study, alexithymic patients were more depressed (p < 10^-3^).

**Conclusions:**

According to our results, depression and alexithymia were found to be significantly inter-related in MS. Future longitudinal studies might better clarify the nature of this relationship in MS patients.

**Disclosure:**

No significant relationships.

